# Authors’ Reply: Statistical inference and effect measures in abstracts of randomized trials, 1975-2021

**DOI:** 10.1007/s10654-023-01081-6

**Published:** 2024-01-04

**Authors:** Andreas Stang, Kenneth J. Rothman

**Affiliations:** 1 Institut für Medizinische Informatik, Universitätsklinikum Essen, Biometrie und EpidemiologieHufelandstr. 55, 45147 Essen, Germany; 2 School of Public Health, Department of Epidemiology, Boston University, 715 Albany Street, Boston, MA 02118 USA

We thank Dr. Macnaughton for the correction [1]. In its official statement [2], the ASA did say that “A *p*-value, or statistical significance, does not measure the size of an effect or the importance of a result.” and “By itself, a *p*-value does not provide a good measure of evidence regarding a model or hypothesis.” The follow-up editorial by Wasserstein et al*.* [3], which indeed was not an official ASA statement, asserted that in the twenty-first century “researchers are free to treat ‘*p* = 0.051’ and ‘*p* = 0.049’ as not being categorically different, [and] … authors no longer find themselves constrained to selectively publish their results based on a single magic number. In this world, … studies with ‘*p* < 0.05’ and studies with ‘*p* > 0.05’ are not automatically in conflict….”

We understand that after teaching and using significance testing for a professional lifetime, some statisticians feel compelled to defend this methodology despite its proclivity to spur misinterpretations of data. We believe that is the reason why many statisticians would prefer to walk back the ASA statement underscoring the problems with significance testing. We emphasize that the p-value itself, while often misinterpreted to mean a probability that chance explains an association, is not the problem. The problem is the conversion of a p-value into a dichotomy of “significant” or “not significant” and the resulting misinterpretation that so often ensues.

**Fig. 1:**
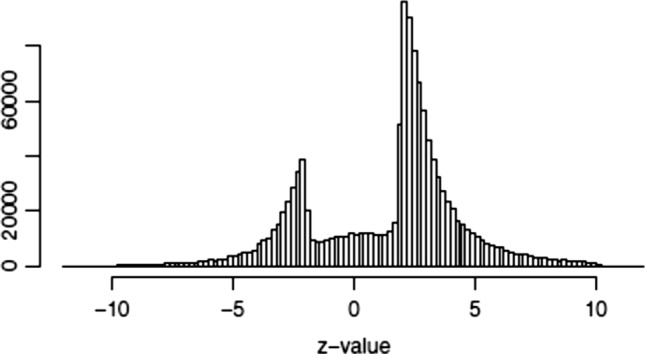
The distribution of more than one million *z*-values from Medline (1976–2019)

A striking illustration of the harm that has been done to science by reliance on statistical significance testing was given by van Zwet and Cator [4] (Fig. 1). They examined the distribution of over 1,000,000 *z*-values from Medline, showing that the distribution is clearly distorted because of the artificial dichotomization of *P*-values in significance testing and the biased publication decisions that ensue. Reliance on significance testing has distorted many individual analyses, and furthermore has had a damaging effect on the literature of science as a whole. Realization of the harm done by significance testing is a crucial reason that many experienced statisticians and researchers regard “statistical significance” as an antiquated tool that is long overdue for replacement (e.g., [5–11]).
